# Protocol for a systematic review of treatment adherence for HIV, hepatitis C and tuberculosis among homeless populations

**DOI:** 10.1186/s13643-020-01470-y

**Published:** 2020-09-13

**Authors:** Luke Johnson, Dan Lewer, Robert W. Aldridge, Andrew C. Hayward, Alistair Story

**Affiliations:** 1 Collaborative Centre for Inclusion Health, Department of Epidemiology and Public Health, University College London, 1-19 Torrington Place, London, WC1E 7HT UK; 2 Department of Primary Care, Population Sciences and Medical Education, University of Southampton, Southampton, SO17 1BJ UK; 3 The Farr Institute of Health Informatics Research, University College London, London, NW1 2DA UK

**Keywords:** Homelessness, Inequality, Inclusion health, HIV, Hepatitis C, Tuberculosis, LTBI, Treatment adherence, Treatment initiation, Treatment completion

## Abstract

**Background:**

Homelessness is a global issue and HIV, hepatitis C and tuberculosis are known to be prevalent in this group. Homeless populations face significant barriers to care. We aim to summarise evidence of treatment initiation and completion for homeless populations with these infections, and their associated factors, through a systematic review and meta-analysis.

**Methods:**

We will search MEDLINE, Embase and CINAHL for all study types and conference abstracts looking at either (1) treatment initiation in a cohort experiencing homelessness with at least one of HIV, hepatitis C, active tuberculosis and/or latent tuberculosis infection (LTBI); (2) treatment completion for those who initiated treatment. We will perform a meta-analysis of the proportion of those with each infection who initiate and complete treatment, as well as analysis of individual and health system factors that may affect adherence levels. We will evaluate the quality of research papers using the Newcastle-Ottawa scale.

**Discussion:**

Given the political emphasis on global elimination of these diseases, and the current lack of understanding of effective and equitable treatment adherence strategies in homeless populations, this review will provide insight to policy-makers and service providers aiming to improve homeless healthcare.

**Systematic review registration:**

PROSPERO CRD42019153150

## Background

The last global survey by the United Nations (UN) in 2005 estimated that 100 million people worldwide were homeless [1]. More recent estimates in 2017 suggest the number is around 150 million people—approximately 2% of the global population [2]. Recent reports show homelessness is growing across almost all European countries [3] and is a significant problem in the USA [4]. With so many people represented within this group, and most with very complex health needs and extremely poor health outcomes [5], homeless health remains an important area of research.

A 2012 systematic review and meta-analysis of prevalence data found studies reporting 0.3 to 21.1% of homeless people have HIV, 3.9 to 36.2% have hepatitis C infection, and 0.2 to 7.7% have tuberculosis [6]. Given the extremely high burden of these communicable diseases, the authors highlighted identifying and managing these infections within homeless health services as a public health priority.

It has previously been shown that a high proportion of homeless people who are diagnosed with one or more of these infections do not either initiate or complete treatment [7–11]. Barriers include mobility among homeless people leading to loss to follow-up; more pressing medical or social needs [7]; lack of attendance to follow-up appointments [12]; stigma and discrimination; on-going alcohol and drug use and criminilisation of these behaviours; lack of stable housing [13]; incarceration; fear of invasive investigations such as liver biopsy; fear of treatment side effects; transport and location of treatment services; lack of flexible appointment times; and problems remembering appointments [14, 15]. With the political emphasis on global elimination of these diseases [16–18], ensuring strategies are in place to overcome these challenges to treatment is essential.

Despite these well-evidenced barriers to care, a systematic review looking at rates of treatment initiation and completion for HIV, hepatitis C and tuberculosis in homeless populations is yet to be undertaken. As well as providing useful data for each infection, such a study could provide understanding as to whether specific factors influence likelihood to adhere to treatment within this cohort. In addition, treatments for these diseases have improved considerably in recent years. Treatment for hepatitis C, for example, has been revolutionised by widespread access to novel direct-acting antiviral treatment (DAAT) [19]. However, understanding the extent to which these benefits have reached the homeless population is lacking.

We aim to perform a systematic review and meta-analysis of treatment initiation and completion rates for HIV, hepatitis C and tuberculosis (both active and latent tuberculosis infection (LTBI)). Collectively, we will refer to treatment initiation and completion as a measure of ‘adherence’. We will analyse papers studying how many homeless people with one or more of these conditions initiate treatment, and/or the number of those who go on to complete treatment. For hepatitis C, we will only include studies using regimens containing DAAT, either as part of treatment or the entire treatment regimen. DAAT requires particular study as it has revolutionised hepatitis C therapy in terms of effectiveness and tolerability and will soon be the mainstay of treatment [19]. For HIV, as there is no end-point to treatment, we will use undetectable viral load as a proxy for sustained treatment adherence instead (which we will categorise as ‘treatment completion’ to parallel other infections). We will include any paper which measures at least one data point for at least one of these diseases.

We will also examine the data for individual and health system factors (as defined later) which are linked to adherence. We hope this data will help enable policymakers and service providers to design more effective and equitable health systems for homeless populations to access treatment for these infections.

## Methods

### Protocol

This protocol is registered with PROSPERO (registration number CRD42019153150) and reported in accordance with guidance provided in the Preferred Reporting Items for Systematic Reviews and Meta-Analyses Protocols (PRISMA-P) statement [20].

### Information source and literature search

We will search MEDLINE, Embase and Cumulative Index to Nursing and Allied Health Literature (CINAHL) for English Language studies from 1990 onwards. 1990 was chosen to balance gathering sufficient papers for analysis with ensuring papers identified are relevant to the challenges faced by homeless populations today. We will also perform hand-searching of the reference lists of included studies, as well as looking up studies which cite our included studies. We will consider all study types and conference abstracts which report homeless population data looking at either (1) treatment initiation in a cohort known or found to have at least one of HIV, hepatitis C, active tuberculosis or LTBI; (2) treatment completion for those who initiated treatment for either HIV, hepatitis C, active tuberculosis or LTBI.

The literature search terms have been designed in collaboration with two experienced health information specialists—one based at University College London, one based at the Norfolk and Norwich Hospital. Full details of the search terms are available in the Appendix.

### Eligibility criteria

We will select studies based on the following criteria:

*Population*—we will define homelessness as adults with no fixed abode (owned or rented), who rely on temporary accommodation, live in institutions or shelters, or live rough (in a context where most people have homes, and homelessness is not because of war, conflict or natural disaster). As we expect to only find a small number of papers meeting our criteria, we will include those in single-room occupancies or marginally-housed within our definition. Study participants must all be currently homeless (for any length of time) and not people with a history of homelessness only. If a subset of a population within a study is homeless, but the data has not been reported separately, we will attempt to contact the authors to obtain homeless-specific data. If they are uncontactable or the data is not available, we will exclude such studies from our review. All homeless people must be clinically diagnosed with at least one of HIV, hepatitis C, active tuberculosis or LTBI either prior to the study or through testing within the study itself. As the type of diagnostic test used will vary, we will accept any individual as having an infection if there is intention-to-treat. We will include homeless people who inject drugs (PWID), but also endeavour to perform an analysis of this group separately within the review if possible due to the unique barriers to care and additional risks of infection and reinfection this group faces. We aim to only look at populations aged 18 years or over. If data contains individuals under 18, we will attempt to contact the authors to obtain data specific to those over 18. If they are uncontactable or data is not available, we will only include samples where the majority of the sample population are over 18; if this is uncertain or not the case, we will exclude such studies from our review. We will look at countries with any income, but will also perform separate analyses based on the World Bank’s divisions of country income [21]).

*Intervention*—we will include any study aiming to treat one or more of the infections. For hepatitis C treatment, we will only include regimens containing DAAT, as previously described.

*Comparison*—we will attempt to compare treatment initiation and completion of HIV, hepatitis C, active tuberculosis and LTBI with the general population in that geographical region. Data for the general population will be gathered from contemporaneous research papers, reports and surveillance data where available.

*Outcome*—our primary outcome will be the proportion of those known or identified as having one of the infections who then initiate and/or complete treatment.

Our secondary aim is to analyse individual and health system factors which could influence adherence levels. We will mark studies including analysis of factors that could contribute to different adherence rates within homeless populations and conduct narrative review of these studies. We anticipate that important individual factors may include history of incarceration, use of injection drugs and mental health problems. We anticipate that health system factors may include the location of treatment services, flexibility of appointment time and programme adherence techniques (including DOTS (Directly Observe Treatment, Short-Course) having a case manager, phone call check-ins, remuneration for patients and housing of homeless patients during treatment). We will be open to including additional adherence techniques we encounter in the literature whilst collating papers.

*Type of study*—we expect most data will be derived from observational, uncontrolled studies but we will consider any study type looking at any aspect of treatment initiation or completion. We will also look at conference abstracts, taking care to ensure they do not duplicate data from published papers. Included studies do not need to cover both treatment initiation and completion, but must report at least one measure of initiation or completion for at least one disease.

### Data collection and analysis

#### Selection of studies

Articles will firstly be double-screened by title and abstract for potentially eligible studies. Any discrepancies will be discussed between the two independent reviewers—if these cannot be resolved, a third reviewer will be involved in the decision. All potentially eligible studies will then be read as full-text papers by two independent reviewers to determine eligibility. Any disagreement which cannot be resolved between the two reviewers will be reviewed by the third reviewer.

#### Data extraction

One reviewer will undertake data extraction with accuracy checked by a second reviewer. A standardised, pretested form will be used to log specific data points for each of the diseases. Any disagreement which cannot be resolved between the two reviewers will again be reviewed by the third reviewer. The data we will collect is detailed in Table 1.

**Table 1:** Data to be extracted from studies included within the review

	HIV	Hepatitis C	Active tuberculosis	LTBI
***Bibliometric data***	Author, year of publication, title of study			
***Study characteristics***	Study design, study funding, country study carried out in, inclusion/exclusion criteria for participants, recruitment method, sample size, any remuneration for participants, location of screening/treatment (e.g. hostel, hospital, mobile screening unit), description of intervention, who is delivering intervention, study start-end date, assessment points, description of any comparison/control (including definition, selection source, any confounding factors identified and/or controlled for)			
***Participant characteristics***	Sex/gender, age, ethnicity, history of imprisonment, comorbidities, reported to be taking injection drugs, non-injection drugs reported to be taking, primary condition(s), history of previously treated hepatitis C or TB			
***Treatment initiation***	Number/percentage who initiated treatment from those referred, reasons for not initiating treatment (if known), type of treatment given			
***Treatment completion***	Undetectable viral load	Number/percentage who completed treatment, method by which treatment adherence/completion measured, programme adherence techniques (e.g. DOTS, case manager, phone calls, housing of patients during treatment), number/percentage lost to follow-up and reasons why if stated (including number found to have died and, if specified, reasons for death)		

#### Data analysis

Our primary aim is to evaluate treatment initiation and completion for each infection. We will report the range of proportions in studies identified, and perform random-effects meta-analyses of proportions to report the average values across studies. We will also perform subanalyses based on the World Bank’s divisions of country income [21].

Our secondary aim is to understand how individual and health system factors influence treatment initiation and completion within homeless populations for each infection. This will be a narrative analysis, and we will consider post-hoc quantitative analysis if there is sufficient data.

We will evaluate the quality of research papers using the Newcastle-Ottawa scale and analyse the data to look for heterogeneity with Cochran’s Q and the *I*^2^ statistic. We also intend to perform sensitivity analyses, dependent on the data we obtain—this will include an analysis based upon research quality. When looking for statistical significance, we will measure effects at the 95% confidence intervals.

#### Strengths and limitations of analysis

To our knowledge, this study will provide the first overview of treatment initiation and completion for important communicable diseases among homeless populations and can inform international policy that aims to eliminate these infections. Homeless populations are likely to experience similar barriers to treatment for all the infections included in this review, and our inclusion of the different infections will allow us to draw on a wider pool of evidence when discussing barriers.

We are aware of several limitations to our analysis. Detail of treatment interventions is likely to vary considerably between studies, and variations in documentation and measurement practices may limit the number of studies that can be included in meta-analysis.

Furthermore, much of the data surrounding individual factors is likely to be collected from questionnaires filled in by participants. Given the sensitivity and stigmatisation of many of the suggested factors, there could be an under-reporting of such risk factors in studies.

Finally, studies are likely to be observational and uncontrolled, limiting the ability to draw causal inferences as to the effect individual and health system factors have on adherence. Pragmatically, however, data will be useful to current health system design and for directing researchers wanting to explore with greater rigour any factors found to affect treatment adherence within this population.

## Discussion

To effectively and equitably treat homeless populations with HIV, hepatitis C and tuberculosis, better understanding is needed as to the proportion of those with each infection who initiate and complete treatment. This review is especially pertinent given the political commitment to global elimination of these diseases [16–18] and recent advances in treatments.

Furthermore, understanding the individual and health system factors which affect adherence will be useful to policymakers and service providers working to improve homeless healthcare, particularly surrounding these infections.

## Data Availability

Not applicable

## Appendix

### Search Terms

The following subject headings are to be included for *homelessness* if present in the database: Transients and Migrants (MEDLINECINAHL)Homeless Youth (MEDLINEEmbase)Homeless Persons (MEDLINECINAHL)Homelessness (EmbaseCINAHL)Homeless Person (Embase)Homeless Woman (Embase)Homeless Man (Embase)Runaways (EmbaseCINAHL)Migrant Worker (Embase). These terms will be combined with a text word search for the following: homeless*. This search was performed in all three databases looking only at the title and abstract of articles

The following subject headings are to be included for *HIV/AIDS* if present in the database: HIV (MEDLINE), HIV infections (MEDLINE, CINAHL), Human Immunodeficiency Virus (Embase, CINAHL), Human Immunodeficiency Virus Infection (Embase), AIDS Virus (Embase), AIDS Victim (Embase), AIDS Sufferer (Embase), AIDS Related Complex (Embase), AIDS Serodiagnosis (CINAHL). These terms will be combined with text word searches for the following: HIV OR human ADJ1 immun* ADJ1 deficiency OR human ADJ1 immun* deficiency ADJ1 virus OR “acquired immune deficiency syndrome” OR AIDS. Searches will be performed in all three databases looking only at the title and abstract of articles.

The following subject headings are to be included for *hepatitis C* if present in the databases: Hepatitis C (MEDLINE, Embase, CINAHL), Hepatitis C Antibodies (MEDLINE, Embase), Hepatitis C Antigens (MEDLINE, Embase), Hepacivirus (MEDLINE), Hepatitis C Antigen (Embase), Hepatitis C Antibody (Embase), Hepatitis C Virus (Embase), Hepatitis C Virus Genotype 1 (Embase), Hepatitis C Rapid Test (Embase). These terms will be combined with text word searches for the following: “hepatitis C” OR HCV. Searches will be performed in all three databases looking only at the title and abstract of articles.

The following subject headings are to be included for *tuberculosis* if present in the databases: Tuberculosis (MEDLINE, Embase, CINAHL), Mycobacterium tuberculosis (MEDLINE, CINAHL), Extensively Drug-Resistant Tuberculosis (MEDLINE), Mycobacterium tuberculosis Complex (Embase). These terms will be combined with text word searches for the following: tuberculosis OR TB OR LTBI. Searches will be performed in all three databases looking only at the title and abstract of articles.

The following subject headings are to be included for *treatment* if present in the databases: therapeutics (MEDLINE, CINAHL), treatment adherence and compliance (MEDLINE), Anti-retroviral agents (MEDLINE), Anti-HIV agents (MEDLINE), Anti-infective agents (MEDLINE, Embase, CINAHL), therapy (Embase), drug therapy (Embase), Medication Compliance (Embase, CINAHL), Antiretrovirus Agent (Embase), Antimicrobial therapy (Embase), Antiviral therapy (Embase). These terms will be combined with text word searches for the following: treatment OR “initiation” OR “completion” OR therapeutics or medication OR “viral suppression” OR “viral load”. Searches will be performed in all three databases looking only at the title and abstract or articles.

The *homelessness* category will then be combined with the AND operator for the *treatment* category, and the AND operator for each of the three disease categories: *HIV/AIDS*, *hepatitis C* and *tuberculosis*. The results of each combination of *homelessness* plus *treatment* plus disease will be combined via the OR operator. Limiters based on publication date (from 1990 onwards) and on language (English only) will then be applied at this stage. We will then review the results of all papers together.

## Abbreviations

CINAHL: Cumulative Index to Nursing and Allied Health Literature
DAAT: Direct-acting antiviral treatment
DOTS: Directly Observe Treatment, Short-Course
HIV: Human immunodeficiency virus
LTBI: Latent tuberculosis infection
PRISMA-P: Preferred Reporting Items for Systematic Reviews and Meta-Analyses Protocols
PWID: People who inject drugs
